# Supplementary material for: Rme1: Unveiling a Novel Repressor in the Cellulolytic Pathway of Trichoderma reesei
Source: J Fungi (Basel). 2025 Sep 6;11(9):658. doi: 10.3390/jof11090658 (PMC12470938; doi:10.3390/jof11090658)
Supplement: Supplementary file 1 [file jof-11-00658-s001.zip › jof-3792173-supplementary.pdf]

Rme1: Unveiling a Novel Repressor in the Cellulolytic Pathway of *Trichoderma reesei*

Antoniêto et al

Supplementary Material

Table S1. Primers used in conventional PCR

Primer	Sequence (5'-3')	Usage
Rme_5'F	GTAACGCCAGGGTTTTCCCAGTCACGACGCACTTCTTCTCTTCCCAAGC	Construction of deletion cassette
Rme_5'R	AACCCAGACAAGACAAGGCAAGTGAGGACTCGTAGCTAATGG	
Rme_3'F	GAAACGGACGGCAACAATCAACCGATCTCTCTTGGGTCACCAGG	
Rme_3'R	GCGGATAACAATTTCACACAGGAAACAGCGTAGTGACTAAACAGCCTCC	
Rme_Pyr4_F	CCATTAGCTACGAGTCCTCACTTGCCTTGTCTTGTCTGGGTT	
Rme_Pyr4_R	CCTGGTGACCCAAGAGAGATCGGTTGATTGTTGCCGTCCGTTTC	
ORF Rme1 F	CAATGGCGATCTCGTCGTATGA	Screening of Δ*rme1* transformants
ORF Rme1 R	ACATCATCTGTCTCGTCCTCCT	
ORF pyr4 F	TGACGGCTTACCTGTTCAAG	
ORF pyr4 R	GATGCCAATCAGCTTGTGCG	
Dir pyr4 F	TGACGGCTTACCTGTTCAAG	
Dir rme1 R	ACCCCTCGTATCGAAACTGA	
Rme1 BamHI F	ACGTGGATCCATGGCGACAGACTGCTTCGT	Cloning
Rme1 XhoI R	ACGTCTCGAGGACCTGCTCCTCGGTGGGGT	
Cel7a F	GTGTAATTTGCCTGCTTGACC	Probes for EMSA
Cel7a R	GGGATCACCAGCTGAGATTG	
Cre1 F	GCGAGGTACAACCGTCTGA	
Cre1 R	TTCGATTCGTAGCATGGGCG	
Swo F	AGCAGCAGCGGCAATAACAA	
Swo R	GCTACATCTGTGGATAGGTAG	

Table S2. Primers used in RT-qPCR

**Gene**	***Foward* (5'-3')**	***Reverse* (5'-3')**
cel7a	CCGAGCTTGGTAGTTACTCTG	GGTAGCCTTCTTGACTGAGT
cel6a	ACAAGAATGCATCGTCTCCG	TGTTCCACCCGTTGTAGTTG
cel7b	CCCTCAACACTAGCCACCAG	AGGTCTTGGAGGTGTCAACG
xyr1	CAATCCTCTCCGTCGCTATTC	CTGTTGCCGAATGTGTTGAC
cre1	CTCCTACTCGTCCTTTGTCATG	GCAAGCATCGTAATGTCGTTG
cel1a	TTTGCCTGGTCGCTCATG	AATCAGCTCGTCAAACAGCG
cel3b	CCAGGATAACTTCAACGA GGG	ATGTGGAGGTTGGAGAACTTG
cel1b	CCATCTACATCACCGAGAACG	TCCAAGTGCGAGTCAAAGTAG
Tr69957	CTCTTACAGTTGGCCCTATCAC	GGTCGGGTTCATAAAGTACGG
xyn2	TGTCAACGAGCCTTCCATC	TCTGCACAGTAACAGTTCCG
cel5a	GCCACTACTATCACCACTTCG	GTACAGCCAAAGTCAAAACCC
cel61a	GCGCCACTGTTCCTGGAG	ACCGCTGCCACCACACTG
cel74a	GCCTTGTATCTGACCTATTCCG	TGATGTCTTTCCAAGTTCCCC
rme1	TGAGGTTGCCGATGACAAG	GGAGAGTAGGTCAGCTTGGAG
actin	TGAGAGCGGTGGTATCCACG	GGTACCACCAGACATGACAATGTT

Table S3. Rme1 amino acid residues and nucleotides in the target promoters involved in the interaction.

Rme1 and Pcre1			Rme1 and Pcel7a		
Position of amino acid residue	Nucleotide position	Lead size (Å)	Position of amino acid residue	Nucleotide position	Lead size (Å)
268	18	4.872	272	21	3.557
276	18	2.91	273	21	3.515
276	19	2.158	273	22	3.973
276	20	4.873	274	20	2.287
277	16	3.777	274	21	2.705
277	17	2.822	274	22	4.403
277	18	4.674	275	21	4.65
306	26	3.854	275	22	4.726
307	25	4.612	276	22	4.728
307	26	4.025	276	23	3.936
309	26	4.664	279	23	4.936
310	26	4.92	280	23	4.047
311	26	4.671	298	21	3.755
312	25	4.194	330	19	4.63
312	26	2.428	330	20	4.2
312	27	2.945	332	19	3.112
325	27	353	332	20	4.459
326	27	4.158	333	18	3.474
327	26	3.576	333	19	2.519
327	27	1.123	336	18	3.335
328	26	3.66	336	19	3.43
328	27	3.312	363	18	3.882
329	25	4.932	364	18	2.464
329	26	3.359	364	19	2.483
329	27	3.812	367	17	4.861
333	26	4.578	367	18	4.301
337	27	4.625	396	16	3.005
369	20	4.733	420	16	1.797
380	21	4.595	420	17	2.693
393	22	3.895			
395	22	4.659			
419	23	4.076			
419	24	1.999			
419	25	4.721			
420	24	2.722			
420	25	2.496			
420	26	4.777			
423	23	4.907			
423	24	2.879			
423	25	1.402			

424	25	4.223			
424	26	3.086			
424	27	2.809			

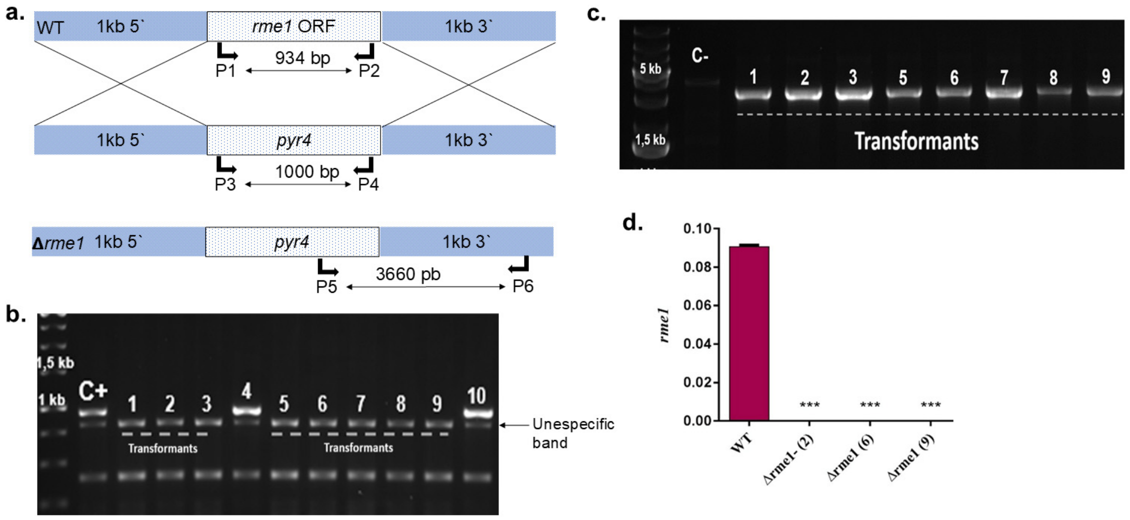

Figure S1. Construction of the Δ*rme1* strain. (A) Schematic representation of the *rme1* deletion in *T. reesei*. The *rme1* ORF was replaced by the selection marker *pyr4* using homologous recombination. (B) PCR using primers P1 and P2 shows the deletion of *rme1* in the candidate transformants. Absence of an amplicon of 1 kb indicates deletion of *rme1* (transformants 1-3 and 5-9). The WT gDNA was used as the positive control. (C) PCR using primers P5 and P6 shows that the deletion cassette was integrated in the right locus in the genome, indicated by an amplicon of 3.6 kb. The WT gDNA was used as the negative control. (D) RT-qPCR showing the absence of *rme1* expression in the selected transformants.

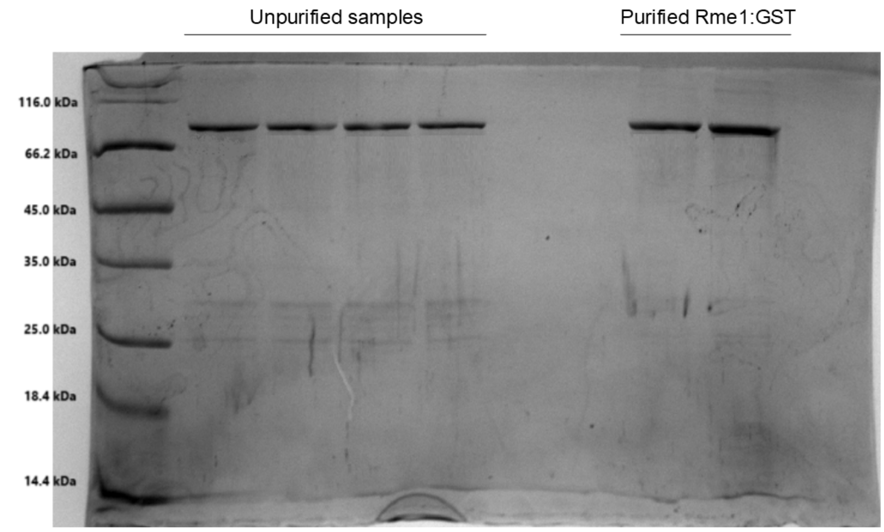

Figure S2. SDS-Page showing the purification of Rme1:GST. Rme1:GST was expressed and produced in *Escherichia coli* Arctic and purified using GST Sepharose 4B GST-Tagged protein purification resin.

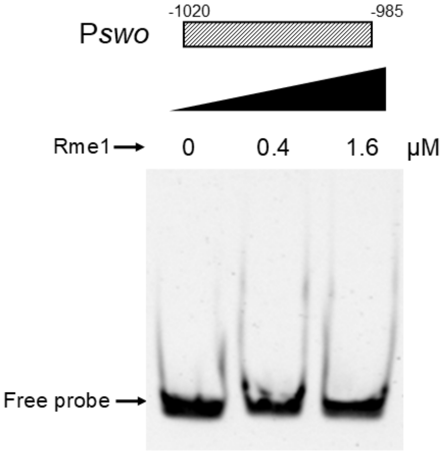

Figure S3. Rme1 doesn't bind to the promoter of *swo* gene. EMSA was performed with increasing concentrations of purified Rme1:GST and a PCR-amplified probe for P*swo* (140 bp).
